# Editorial: Advancing performance: biomechanics in paralympic and adapted sports

**DOI:** 10.3389/fspor.2025.1683091

**Published:** 2025-08-29

**Authors:** Lorenzo Rum, Rafael Kons

**Affiliations:** ^1^ Neuro Ortho Research Movement Analysis Lab, Department of Biomedical Sciences, University of Sassari, Sassari, Italy; ^2^ Department of Physical Education, Faculty of Education, Federal University of Bahia, Salvador, Brazil; ^3^ Human Physiology and Sports Physiotherapy Research Group, Vrije Universiteit Brussel, Brussels, Belgium

**Keywords:** biomechanics, paralympic sports, adapted sports, athletic performance, assistive technology, injury prevention, performance optimization, evidence-based classification

**Editorial on the Research Topic**
Advancing performance: biomechanics in paralympic and adapted sports

## 1 Introduction

The field of biomechanics is fundamental to advancing both performance and the safety of Paralympic and adapted sports athletes. As the Paralympic movement continues to grow and evolve, so does the need for sport- and impairment-specific research that addresses the biomechanical challenges faced by athletes with disabilities (1, 2). By understanding the interaction between the biomechanical principles underlying human movement and impairment, researchers can drive the progress in the development of innovative strategies to optimize performance, prevent injuries and improve the design of assistive technologies and evidence-based classification systems in Paralympic Sports (3–7).

This Research Topic aims to explore these various aspects of biomechanics in Paralympic and adapted sports, providing a platform for disseminating innovative research. The articles gathered illustrate how biomechanical, physiological and functional assessments can inform both individualized performance and training strategies as well as classification criteria.

## 2 Sport- and impairment-specific assessment of performance

Several contributions focus on understanding sport-specific performance factors and their implications for classification and training. In Altmann et al., inertial measurement units (IMU) were used to assess standardized wheelchair mobility and ball-handling tasks in wheelchair rugby athletes with coordination impairments (CI) and those with other impairments. The study aimed to create a standardized performance framework for athletes with CI, a group underrepresented in the current classification model. While overall performance did not significantly differ between impairment types, more pronounced challenges emerged for CI athletes in tasks requiring precise hand placement and combined wheelchair and ball activities. The study suggests that impairment-specific tools are still needed to better capture functional limitations in this group.

Similarly focusing on wheelchair sport, Honnorat et al. examined age-related effects on sprint performance in wheelchair basketball. Fourteen junior and eight senior athletes completed repeated sprints with IMU-based kinematic analysis. Seniors displayed greater velocity and less fatiguability, but cluster analysis revealed that factors like experience and wheel size may influence performance more than age alone, especially in small and heterogeneous samples.

Adding a cross-sport comparison, Brassart et al. explored interlimb asymmetry and fatigue responses in repeated sprint tests among wheelchair basketball and rugby players using IMUs. Asymmetry remained stable despite fatigue, suggesting it is not a key contributor to performance decline. Basketball players generally demonstrated better performance than rugby athletes, but only the latter showed significant fatigue effects. These results reinforce the need for sport-specific performance benchmarks and underline differences in fatigue profiles linked to impairment types and tactical demands.

## 3 Individualized approach through case studies

In parallel, other studies adopted a subject-specific lens, emphasizing the importance of individualized analysis in Paralympic sports, where impairment heterogeneity is often high. Quittmann et al. presented a detailed case series on elite handcyclists, using synchronized measurements of kinetics, kinematics, and surface EMG to uncover individualized propulsion strategies under various exercise modalities. The observed variability in work distribution, joint motion and muscle activation patterns underscores the inadequacy of one-size-fits-all approaches in both training and classification.

A similar individual approach was used by Antunes et al., who tracked a T36 sprinter with cerebral palsy over two competitive seasons using vertical jump tests. Weekly assessments revealed strong correlations between jump metrics and sprint performance (particularly in 100 m and 200 m), with the athlete remaining injury-free. This longitudinal case supports vertical jumps as a practical tool for load monitoring and performance tracking in athletes with coordination impairments.

Holdback et al. investigated seated shot put performance in an athlete with CI by testing three pole grip heights using an instrumented pole and motion capture. Trunk angular velocity and power were sensitive to grip position, and the configuration maximizing these variables was associated with potential improvements in throwing performance over four weeks. While limited to one athlete, the study offers a personalized framework for technical optimization in seated throwing events.

## 4 Muscle biomechanics for training optimization

Linking subject-specific observations to training adaptation, the final paper examined the impact of high-velocity training on muscle architecture and function. Gallinger et al. evaluated the effects of a 10-week high-velocity training program in ambulatory adults with cerebral palsy. The study found a significant increase in resting fascicle length of the medial gastrocnemius, but no group-level changes in muscle function. However, five out of eight participants showed individual improvements, pointing to high inter-individual variability and the importance of personalized responses to training. Despite the absence of clear functional gains, the protocol provides valuable guidance for clinicians and strength and conditioning professionals working with high-functioning individuals with CP.

## 5 Conclusion and future directions

Together, the articles in this Research Topic demonstrate the value of biomechanical assessment in Paralympic and adapted sports, whether for individualized monitoring, training optimization, or informing classification procedures. However, key challenges remain. First, while small samples allow for detailed individualized analyses, they inevitably limit the statistical power and generalizability of findings. In Para sports where impairment types, training histories and equipment configurations vary widely, these constraints make it difficult to establish standardized performance benchmarks or classification criteria. Second, longitudinal monitoring remains uncommon (only the T36 sprinter case in this Research Topic incorporated regular, season-long assessments). Extending such monitoring across athletes, sports and impairment groups could provide valuable insights into how performance evolves over time. Third, biomechanical assessments are still too often performed in isolation from physiological data. Integrating measures such as metabolic cost or heart rate parameters could yield richer and more holistic performance models, enabling a deeper understanding of impairment impact on performance.

Finally, a notable trend in this Research Topic is the growing focus on motor coordination impairments, a group often underserved in classification and research. By embracing both sport-specific and individualized approaches, the studies here contribute to a more precise, evidence-based future for Paralympic sport. As biomechanics continues to bridge the gap between impairment and performance, this body of work advances the shared goals of fairness, inclusion, and excellence in adapted sport.
